# p53-Dependent Repression: DREAM or Reality?

**DOI:** 10.3390/cancers13194850

**Published:** 2021-09-28

**Authors:** Sylvain Peuget, Galina Selivanova

**Affiliations:** Department of Microbiology, Tumor and Cell Biology, Karolinska Institutet, SE-171 76 Stockholm, Sweden

**Keywords:** p53, transcription, gene repression, signal integration

## Abstract

**Simple Summary:**

The tumor suppressor p53 is a complex cell signaling hub encompassing multiple transcription programs and governs a vast repertoire of biological responses. However, despite several decades of research, how p53 selects one program over another is still elusive. Recent attempts have used meta-analyses of p53 ChIP-seq data to determine the core p53 transcriptional program, conserved across different models and stimuli. This review highlights the complexity of the multiple layers of p53 regulation and the context specificity of p53 target genes. More specifically, we discuss the controversy over the mechanisms of p53-dependent transcriptional repression and its potential role in the flexibility of p53 response.

**Abstract:**

p53 is a major tumor suppressor that integrates diverse types of signaling in mammalian cells. In response to a broad range of intra- or extra-cellular stimuli, p53 controls the expression of multiple target genes and elicits a vast repertoire of biological responses. The exact code by which p53 integrates the various stresses and translates them into an appropriate transcriptional response is still obscure. p53 is tightly regulated at multiple levels, leading to a wide diversity in p53 complexes on its target promoters and providing adaptability to its transcriptional program. As p53-targeted therapies are making their way into clinics, we need to understand how to direct p53 towards the desired outcome (i.e., cell death, senescence or other) selectively in cancer cells without affecting normal tissues or the immune system. While the core p53 transcriptional program has been proposed, the mechanisms conferring a cell type- and stimuli-dependent transcriptional outcome by p53 require further investigations. The mechanism by which p53 localizes to repressed promoters and manages its co-repressor interactions is controversial and remains an important gap in our understanding of the p53 cistrome. We hope that our review of the recent literature will help to stimulate the appreciation and investigation of largely unexplored p53-mediated repression.

## 1. Introduction

The transcription factor p53 is the most frequently mutated gene in cancer and arguably the most critical barrier against tumorigenesis. Because of its crucial importance in most types of cancer, it has become one of the most studied genes [1]. The high potency of p53-mediated tumor suppression has encouraged intensive efforts to target the p53 pathway by small molecules or peptides to restore its function for anticancer therapy. Many of these are starting to be translated into clinics and are currently undergoing clinical testing [2,3].

While transcription-independent functions of p53 that participate in its pro-apoptotic properties have been described [4], the primary function of p53 is its activity as a transcription factor. Substantial evidence obtained by different labs allows us to conclude that p53, rather than triggering a global stress- and cell-type invariant response, may tune the transcriptional response in lineage- and signal-specific manner, culminating in a broad range of biological responses (reviewed in [1]). In this view, p53 functions as a hub of cell signaling that integrates multiple stress signals (such as oncogenic stress, replication stress, DNA damage, oxidative stress, hypoxia or ribosomal stress) into an appropriate, context-dependent, transcriptional response. However, we still have much to learn to direct p53 towards the desired outcome (i.e., cell death, senescence or other) selectively in cancer cells without suppressing normal tissues or the immune system.

Recently, several studies tried to simplify the complexity of the p53 response toward its core target genes by performing a meta-analysis of p53 cistrome and transcriptome in multiple cell lines upon different stimuli. The authors of these studies suggest that the direct p53 core genes involve a certain number of activated target genes, while p53-dependent repression is only indirect, occurring via induction of p21 followed by activation of the downstream DREAM repressive complex (DP, Rb-like, E2F4 and MuvB) [5,6,7]. In this review, we highlight the contextuality of the p53 response discarded by these studies. In particular, we discuss why signal- and lineage-specific transcriptional programs and p53-repressed genes, which meta-analysis could ignore, may be the key to understanding the multiple faces of p53.

## 2. Factors Which Confer the Diversity of p53 Responses

p53 induces a remarkable variety of biological responses, ranging from transient or permanent growth arrest (senescence), induction of pro-oxidant response and apoptotic cell death [8], modulation of immune response [9], inhibition of metastatic potential/plasticity of cancer cells [10] or block of angiogenesis [11], regulation of autophagy [12] or iron-dependent form of cell death (ferroptosis) [13], to stimulation of DNA repair [14] and anti-oxidant responses [15], as well as control of metabolism [16] and repression of pluripotency [17]. The choice of transcriptional program induced by p53 is dictated by a number of cooperating and antagonizing factors and their combinations, making it an overwhelming task to predict the p53 response in different settings.

Reactivation of the p53 killer function (i.e., apoptosis) in established cancers is the ultimate goal of p53-based therapies. However, the induction of p53-mediated apoptosis in normal tissues is the cause of toxicity upon chemotherapy and the pathological loss of cells in neurodegenerative diseases, ischemia and stroke. Induction of senescence by p53 might also contribute to aging [18]. On the other hand, p53-dependent triggering of growth arrest and DNA repair in cancer tissues upon radio-and chemotherapy can lead to cancer recurrence and resistance to therapy. The induction of senescence by p53 is also controversial, as it has been shown to impede the response to chemotherapy in breast cancer patients [19]. However, in patients with acute promyelocytic leukemia (APL), the induction of senescence by p53 is beneficial [20]. The outcome might be determined by the type of factors secreted by senescent cells, which defines the type of immune cells recruited to a tumor. Thus, it is clear that, in order to successfully apply p53-based therapies, we need to thoroughly investigate the mechanisms by which p53 induces its vast range of responses.

**Figure 1:**
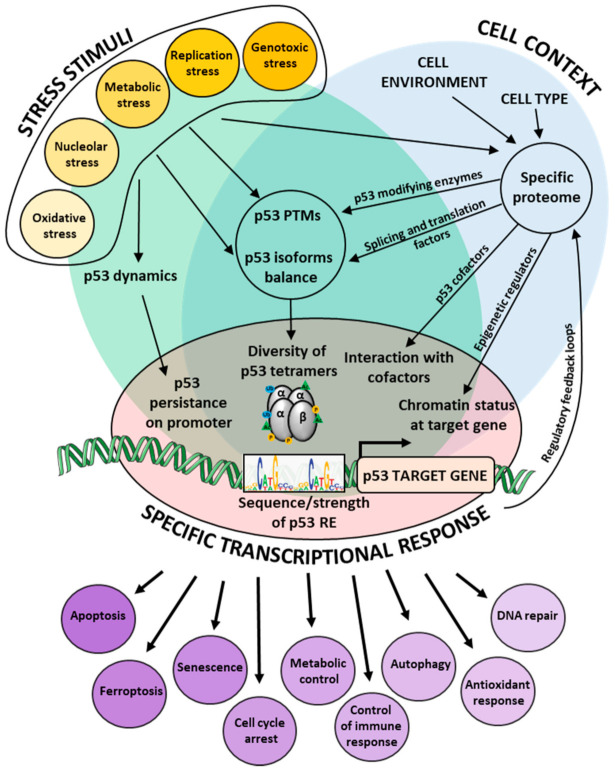
p53 is tightly regulated at multiple levels in the cells, including its dynamic expression, PTMs, isoforms balance, cofactor binding and others. This creates a great diversity in p53 complexes on its target promoters, depending on the type of p53-activating signal and the cellular context. Together with the specific chromatin state, all these factors contribute to fine-tuning of p53 response, thus ensuring the adaptability of its transcriptional program.

p53 is a transcription factor that binds to its response elements (RE) in target genes and facilitates or prevents the formation of active transcription complexes, thus activating or repressing gene expression. p53 transcriptional activity is tightly regulated at multiple levels (Figure 1).

p53 is heavily regulated by more than 300 different posttranslational modifications (PTMs), such as phosphorylation, acetylation, ubiquitination, neddylation, SUMOylation and methylation (reviewed in [21]). PTMs have long been shown to regulate the choice of p53 transcriptional program. For instance, p53 acetylation-deficient mouse models have demonstrated the importance of the acetylation status of the lysines in the p53 DNA binding domain (Lys101, Lys120, and Lys164) for p53 target selectivity [22,23]. Acetylation of Lys164 was shown to drive the transcription of cell cycle-related genes, while Lys101 acetylation is crucial for inducing ferroptosis. Mechanistically, PTMs control p53 stabilization and activation upon stress, its conformation and interactions with cofactors or DNA, thus modulating p53-induced transcriptional programs and biological outcomes. For instance, transactivation of pro-apoptotic targets is facilitated by HIPK2-mediated phosphorylation of p53 at Ser46 or by acetylation at Lys120 by Tip60 [24,25]. While binding to ASPP promotes apoptosis [26], Hzf1, Brn3A and hnRNPK have been shown to cooperate with p53 in the induction of genes involved in growth arrest [18,27]. In turn, BRCA1 and Ref-1 direct p53 towards the induction of DNA repair genes [28,29]. However, despite the numerous studies that looked into the network of p53 regulators (reviewed in [18,30]), it remains elusive how they guide p53 to a certain transcriptional program, and even more importantly, how they manipulate them.

On top of PTM complexity, the TP53 gene can give rise to twelve different protein isoforms by a combination of alternative splicing, usage of IRES and alternative promoter [31]. All p53 isoforms include the p53 oligomerization domain and therefore could be incorporated into p53 tetramers. However, the N-terminal-truncated isoforms ∆133p53 and ∆160p53 have an altered DNA-binding domain, while ∆40p53 isoforms lack the first transactivation domains, thus changing their DNA binding properties in both cases [32]. Several studies revealed that the balance of p53 isoforms indeed influences the composition of p53 tetramers and impacts p53 transcriptional activity and promoter selectivity. For instance, it has been shown that ∆40p53 isoforms induce a transcriptional program different from that of full-length p53 [33]. Similarly, ∆133p53 isoforms change the promoter selectivity of p53, thereby modifying the p53 transcriptional response and cellular outcome [34,35].

Another level of regulation is the persistence of p53 on the promoter. Several studies have highlighted the importance of p53 dynamics for its transcriptional response [36,37,38]. As p53 activates the expression of its negative regulators, such as MDM2 and WIP1, it leads to oscillations in its protein level. Time-course ChIP-seq experiments have demonstrated a similar pulsatile dynamic of p53 binding on its target promoters [38]. Converting these p53 pulses into a sustained signal, for instance upon MDM2 inhibition, changes the p53 transcription profile and affects cell fate decision [36,37].

Overall, a network of multiple factors affects p53 transcriptional response, but our understanding of p53 life or death decisions constitutes only the tip of the iceberg. More systematic studies are required to address these urgent questions of p53 biology and provide new ideas for combination therapies to direct p53 response to the desired outcome.

## 3. Identification of the Core Transcriptional Program of p53

Our analysis of genome-wide chromatin occupancy by p53 using ChIP-seq revealed the “p53 default program” (i.e., a similar pattern of p53 binding to chromatin in MCF7 breast cancer cells), irrespective of the activating compound (nutlin3a, RITA or 5-FU) and regardless of distinct transcriptional and biological responses triggered by these compounds [39]. The core set of *ca* 100 p53 target genes have been recently defined by comparing multiple complementary datasets. These are induced irrespective of PTMs and biological outcomes (growth arrest/senescence) upon nutlin treatment in three cancer cell lines of different origins [7]. The core set of p53 target genes contains diverse biological functions, such as apoptosis, cell cycle arrest, differentiation, metabolism, p53 control, autophagy and DNA repair. This discovery provides p53 researchers with a valuable tool to address the role of p53 in different biological processes. However, it would be naïve to suppose that the core genes can explain all aspects of p53 biology, such as modulation of immune response, inhibition of angiogenesis and metastasis, pluripotency and cell plasticity [1,7]. Moreover, a recent work by Moyer et al. has demonstrated in vivo that there is an extremely low number of genes targeted by p53 regardless of context [40]. The authors compared the p53 transcriptional response in different organs using a genetically engineered mouse model of p53 activation mediated by conditional deletion of Mdm2. Only seven p53-induced genes were common between all organs (*Ccng1*, *Eda2r*, *Gtse1*, *Mdm2*, *Polk*, *Psrc1*, *Zfp365*), while the canonical p53 target gene *p21* was not among them. This extreme context specificity of the p53 response illustrates how the definition of the core p53 program is challenging and may be biologically irrelevant in a broader picture.

Overall, considerable evidence supports the notion that the diverse p53-mediated transcriptional programs are governed by the epigenetic state of the chromatin and thus the availability of p53 RE, its PTMs and the repertoire of co-regulators, which greatly vary among cell types and upon different stresses [41,42].

Thus, genes involved in ‘non-core functions’, including p53-repressed genes, most probably determine the extraordinary flexibility of the context-specific p53 response and drive its tumor suppressor properties in different tissues.

## 4. Controversy over the Mechanisms of p53-Mediated Repression

**Figure 2:**
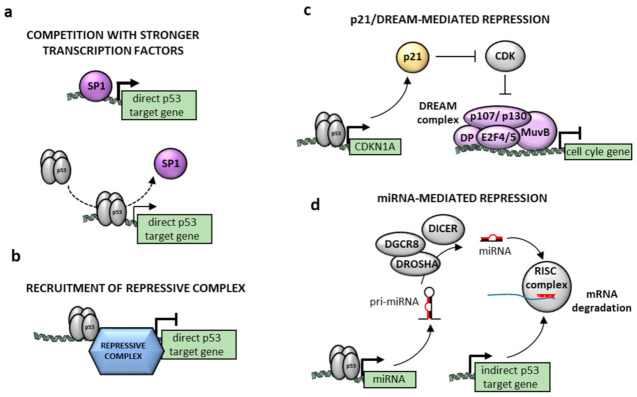
Main possible mechanisms of p53 dependent repression: (**a**) Direct repression by p53 can be caused by the displacement of a transcription factor by p53 binding on the promoter of its target, such as SP1. (**b**) Alternatively, p53 could interact with specific cofactors to recruit a repressive complex, leading to local chromatin rearrangement and loss of accessibility to the promoter of the target. (**c**,**d**) Indirect repression by p53 is caused by the activation of a p53 target gene which in turn inhibits its target’s transcription or mRNA stability. In the case of the DREAM complex (**c**), p53-dependent transcription of p21 inhibits CDK, which prevents the phosphorylation of the Rb family members p107 and p130. Hypophosphorylated p107/p130 then forms the repressive DREAM complex to restrict the expression of genes with upstream cell cycle-dependent element (CDE) or cell cycle genes homology region (CHR) promoter sites. Transcriptional repression is also achieved through p53-dependent transcription of microRNAs, such as miR34a (**d**).

The molecular mechanism of p53-mediated gene repression is a subject of active debate (Figure 2). p53 can repress genes indirectly via its target genes p21, E2F7, microRNAs (miR34a) and long non-coding RNAs (Pvt1b or lincRNA-*p21*). The direct repression by p53 involves binding to the gene and recruitment of co-repressors/HDACs or competition with/sequestration of transcriptional activators (reviewed in [43]). Despite intensive research focused on the mechanisms of p53-mediated repression, it remains to be elucidated how p53 activity is switched from activation to repression. Several factors have been suggested to cooperate with p53-mediated repression, including NF-Y, mSin3a, HDAC1 and others (reviewed in [43,44]). Antagonistic interaction (i.e., competition) with SP1 has been reported, for example, for the repression of genes encoding telomerase and nestin [45,46].

Another interesting possibility is that p53-dependent repression could be mediated by epigenetic silencing. Indeed, p53 has been shown to regulate the transcription of the DNA methyltransferases DNMT1, DNMT3a and DNMT3b, and the TET enzymes Tet1 and Tet2; hence, p53 can regulate DNA methylation homeostasis [47]. Furthermore, p53 cooperates with DNA methylation to restrict the expression of retrotransposons [48]. The exact mechanism of the retrotransposon’s repression is still unknown, but interestingly, several 53 binding sites, accounting for up to 30% of the p53 binding sites in the genome, have been identified in the promoters of endogenous retroviruses [49]. As p53 has also been shown to directly interact with DNMTs [50,51], it is possible that p53 recruits methyltransferases to silence endogenous retroviruses.

A gene is regarded as a direct p53 target if it matches at least three of the four criteria: (1) p53-dependent differential expression; (2) the presence of p53 response element (RE) in the promoter; (3) p53 binding to its RE; (4) p53-dependent regulation of the Luc reporter containing p53 RE from the gene. Based on these strict criteria, at least two dozen p53-repressed genes have been defined as direct targets [8]. A few examples of recently identified and thoroughly validated p53-repressed targets include genes encoding fibrillarin [52]; cancer-associated lipogenic enzyme SCD [53]; nestin, connecting p53 with cellular plasticity [46]; PINK1, which links nuclear p53 to the modulation of mitophagy [54]; c-Myc [55]; and IKKβ [56]. Additionally, p53 has been shown to directly bind the promoter and repress the expression of LINE1 retrotransposons by stimulating local deposition of repressive histone marks at these transposons by a still unknown mechanism [57]. Importantly, this function of p53 to restrict mobile elements by closing the chromatin is conserved in *Drosophila* and zebrafish models, demonstrating its importance during evolution [58].

However, in recent years, several studies have suggested that p53 represses genes solely indirectly through the induction of CDK inhibitor p21, which prevents phosphorylation of Rb and Rb-like proteins, thus transmitting the signal from p53 to a repressor DREAM complex [5,6,59] (Figure 2c). Among genes repressed in a p53-dependent manner, the authors identified only cell cycle-associated genes regulated by the p53-p21-DREAM-E2F/CHR pathway. This conclusion relies on the meta-analysis of ChIP-seq, and transcriptome data obtained in previous studies in different cell lines upon different stimuli.

The major limitation of meta-analysis is that it considers only those peaks and genes that are reproduced in all data sets, disregarding lineage- and stress type-specific gene regulation by p53—since these, by definition, are not common for all datasets, and therefore appear to have lower p53 signal in meta-analysis. Thus, by no means does this meta-analysis of different datasets identify the whole p53 cistrome and transcriptome. These studies have identified a core, or default, p53 chromatin occupancy pattern and transcriptional program, which are triggered in all types of cells irrespective of cellular chromatin state, the nature of activating signal, p53 PTMs and diverse biological outcome. As mentioned above, all these factors create the diversification of context-dependent p53 transcriptional regulation, which needs to be investigated to enable us to manipulate p53 for a long cancer-free life.

## 5. Low p53 Occupancy on Repressed Genes Might Be Due to a Short Time of Residence

**Figure 3:**
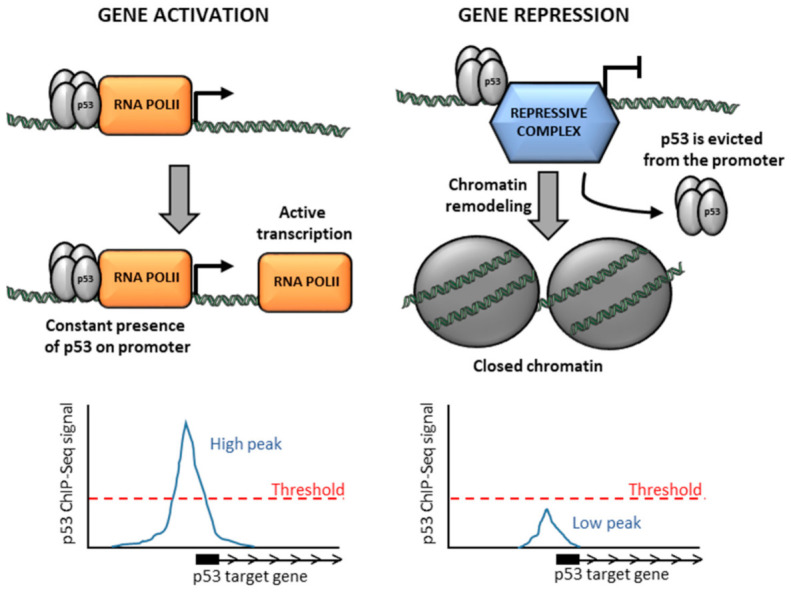
Shorter time of residence of p53 at activated versus repressed promoters. Activation of transcription requires the continuous presence of a transcription factor (i.e., p53) at the promoter to ensure accessibility of chromatin for transcription machinery and unceasing recruitment of initiating RNA polymerase to replace elongating RNA polymerase complexes. On the contrary, once the repressive chromatin state at the promoter is established, it is self-maintained in the absence of inducing signal and does not require the constant presence of the initial repressor (p53), leading to a short residence time of p53 at the repressed promoter, which is reflected as lower occupancy of p53 in cell population-based ChIP-seq analysis.

We have previously analyzed p53 occupancy at repressed genes and noted that p53 peaks are usually smaller on repressed genes in comparison to activated ones [39]. We suggest that the low occupancy of p53 at repressed promoters most probably reflects a fewer number of cells in which p53 is bound to these promoters at a given moment (i.e., a more transient binding mode of p53 to repressed genes). The transient binding of p53 to repressed promoters might reflect a different mode of transcriptional repression versus activation. Activation of transcription requires the constant presence of an activator to ensure an open chromatin structure accessible for transcriptional machinery and continuous recruitment of RNA polymerase. In contrast, once the repressive chromatin state at the promoter is established, it is self-maintained in the absence of an inducing signal due to initial modifications providing binding sites for the enzymes that keep chromatin closed. Therefore, transcriptional repression does not require the initial transcription factor [60], leading to a shorter residence time of the transcription factor on the repressed promoter (Figure 3).

## 6. Low Affinity p53 REs

A comprehensive meta-analysis of ChIP-seq data from different experiments remains a challenge; as such, most computational methods consider only the top few hundred highest peaks with the highest affinity RE, whereas low peaks/weaker RE are completely ignored. Nevertheless, several studies have highlighted the role of low-affinity REs for robustness, specificity and precision of gene regulation (reviewed in [61]).

Previous studies (reviewed in [8]), as well as our ChIP-seq analysis [39], suggest that p53-repressed genes frequently display lower affinity RE in their promoters. Another direct comparison of ChIP-seq data from different cells treated with several p53-activating compounds revealed that the ‘default set’ of p53 binding sites common to most cell types and independent of the type of activating stimuli largely comprises high affinity p53 RE. In contrast, RE that was bound by p53 in a cell-type and stress-specific manner was enriched in low-affinity sites, suggesting its importance in fine-tuning the p53 response in a context- and signal-specific manner [62].

Notably, analysis of the p53 REs sequence in repressed genes showed more flexible dinucleotide combinations in the core CWWG of p53 RE compared to p53-activated genes [63]. Importantly, genes with low-affinity binding sites play a significant role in p53-mediated tumor suppression, as their expression correlates with superior survival in breast cancer patients [62].

Interestingly, the lower affinity binding sites might require a higher level transcription factor to achieve a productive binding, as shown for another transcription factor—c-Myc, which can activate and repress genes. c-Myc occupancy at repressed genes is lower than that at activated genes and requires higher concentrations of c-Myc for transcriptional repression [64]. This implies that smaller p53 peaks, in addition to being lineage- and stress type-dependent, might represent lower affinity binding sites in repressed genes; therefore, regulation of these could be achieved only at later time points, upon maximal accumulation of p53. Such genes could be overlooked by analyzing only early p53-bound genes by GRO-seq before the p53 level rises [65]. As it has been pointed out in this study, the known p53 target pro-apoptotic genes, which have low-affinity RE, were not affected by low levels of p53 at early time points. The same might refer to the repressed genes.

Recently, a thorough meta-analysis of 46 p53 ChIP-seq datasets has found that only 14% of genes bound and repressed by p53 are DREAM targets [66]. By considering all datasets independently and not only their intersection, p53-bound and -repressed genes were shown to be highly dependent on the type of stress stimulus and cellular context, with 3% of the repressed genes even being upregulated in another context [66]. These results support the idea of the involvement of p53-repressed genes in the adaptability of p53 transcriptional programs.

## 7. Concluding Remarks

In summary, to identify genes involved in the diversification of the p53 transcriptional response in a signal- and lineage-dependent manner, we need to consider lower affinity/lower occupancy p53 binding sites, including those in p53-repressed genes. More systematic studies are required to elucidate the difference between the p53 RE in activated versus repressed genes. It is possible, for example, that the presence of another transcription factor at the promoter due to a composite binding site (i.e., site overlapping with p53 RE) can switch p53 activity towards transcriptional repression. Interestingly, our bioinformatics search for transcription factors with composite binding sites in p53-repressed promoters has identified several of those, including E2F family members (unpublished data). We speculate that activated p53 and E2F bound to hypophosphorylated Rb might cooperate to repress genes. Further studies will hopefully help clarify these issues and identify cellular cofactors and small molecules that can switch p53 activity from gene activation to repression in a controlled fashion.
